# Initiation of enteral and parenteral feeding and why we interrupt them in the critical care setting

**DOI:** 10.1186/2197-425X-3-S1-A191

**Published:** 2015-10-01

**Authors:** AKA Abi Musa Asa'ari, ST Passey, B Carr

**Affiliations:** Critical Care Unit, University Hospitals of North Midlands Trust, Stoke-on-Trent, United Kingdom

## Introduction

Initiation and appropriate delivery of nutrition support is fundamental in the care of critically ill patients. To optimise delivery of the prescribed energy and protein there is the added challenge of minimising the frequency and duration of interruptions in order to meet nutritional needs.

## Objectives

We explored the current practice of our CCU looking specifically at time to initiation of feed; and interruptions to feed once started.

## Methods

We collected retrospective data from 50 consecutive CCU patients receiving ≥7 days of enteral/parenteral feed from July to November 2014. Data included; time from CCU admission to initiation of feed and interruptions over the first 7 days including type, duration and frequency. We compared our practice with guidelines that suggest feed should be started within 48 hours. [1, 3]

## Results

66% of our patients were started on a regime within 48 hours of their CCU admission. 34% started their feed after >48 hours.

We found 8 types of interruption, with 4 being more frequent. The most common was related to nasogastric (NG) medications. The second was due to poor absorption, indicated by high aspirates and vomiting. Imaging and procedures came after. We calculated the mean duration for each interruption type. This showed high vasopressor requirement caused the longest interruption, however this pertains to only 2 patients. Absorption issues were the next longest duration of interruption with a mean time of 9.65 hours. We found a large amount of interruptions not documented.

Days 1 and 2 of feeding were the least interrupted. Day 5 was the mode and 12% of patients managed 7 uninterrupted days of enteral/parenteral feed.

## Conclusions

66% patients started feed within the first 48 hours. 6 patients waited >72 hours, to a maximum of 131 hours. This is an area we need to improve on. NG drug administration was the most common cause for interruption; this was resolved by increasing the rate to achieve the same total in fewer hours. More interventions are needed to resolve issues regarding poor absorption of feeds and interruptions due to delayed procedures. 19.7% of interruptions had no documented cause; this is an area for improvement for nursing/medical staff. Only 12% managed 7 days of non-interrupted feed half of them were on parenteral nutrition. Day 5 was the most interrupted day due to it being the day most likely for patients to be extubated or tracheotomised.

**Figure 1:**
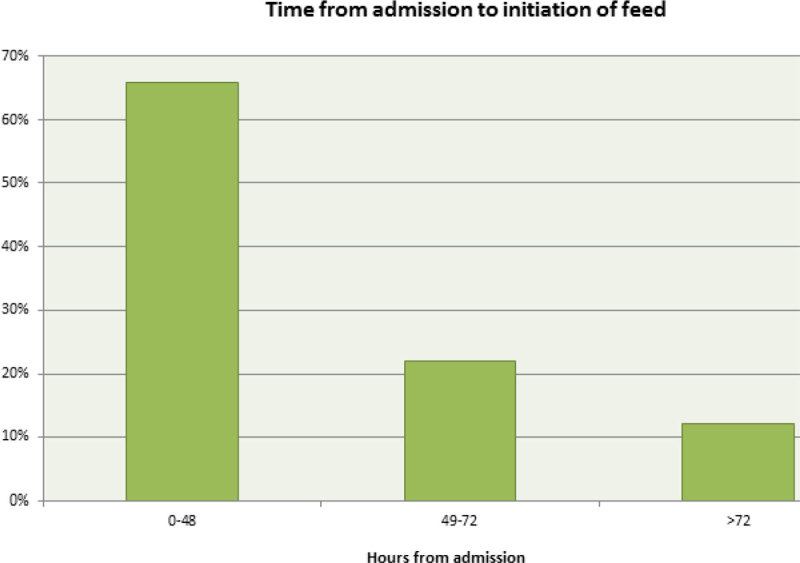
Figure 1

**Figure 2:**
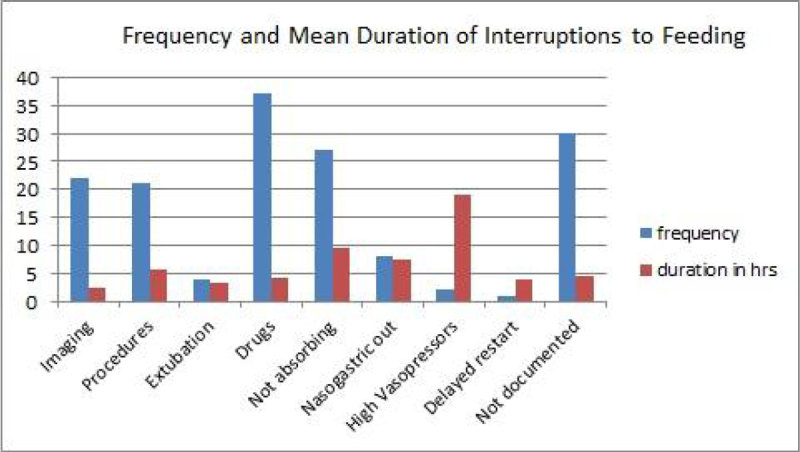
Figure 2

**Figure 3:**
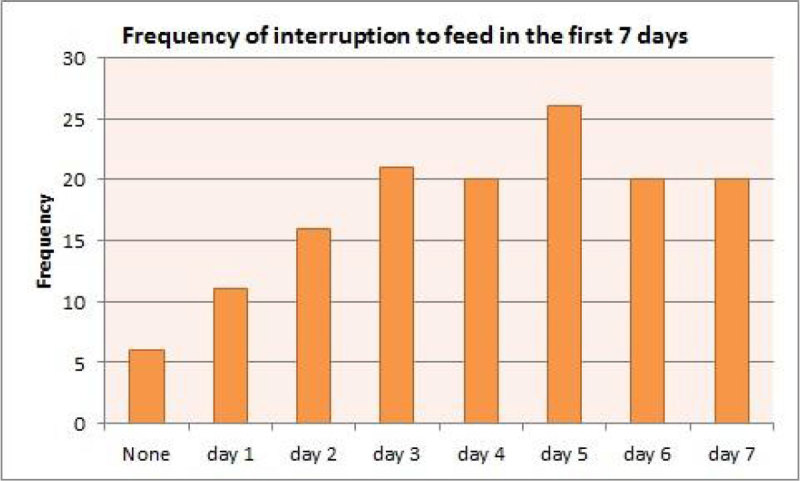
Figure 3
